# Supplementary material for: Mechanisms governing the pioneering and redistribution capabilities of the non-classical pioneer PU.1
Source: Nat Commun. 2020 Jan 21;11:402. doi: 10.1038/s41467-019-13960-2 (PMC6972792; doi:10.1038/s41467-019-13960-2)
Supplement: Supplementary file 7 — Source data [file 41467_2019_13960_MOESM7_ESM.zip › Source_Data/Figure6/Figure6B_MotifScanOutput/ETS1_mutPU.1/homerResults/motif58.similar.html]

motif58

## Information for motif58

T
G
A
C
C
A
G
T
T
G
A
C
A
C
G
T
G
T
A
C
A
C
T
G
T
G
A
C
A
T
C
G
T
G
C
A
A
T
C
G
C
T
G
A
  
Reverse Opposite:  

G
A
C
T
T
A
G
C
A
C
G
T
A
T
G
C
A
C
T
G
T
G
A
C
A
C
T
G
T
G
C
A
A
C
T
G
G
T
C
A
A
C
T
G
  

|  |  |
| --- | --- |
| p-value: | 1e-14 |
| log p-value: | -3.451e+01 |
| Information Content per bp: | 1.720 |
| Number of Target Sequences with motif | 102.0 |
| Percentage of Target Sequences with motif | 0.76% |
| Number of Background Sequences with motif | 80.9 |
| Percentage of Background Sequences with motif | 0.23% |
| Average Position of motif in Targets | 105.8 +/- 57.3bp |
| Average Position of motif in Background | 113.5 +/- 60.4bp |
| Strand Bias (log2 ratio + to - strand density) | -0.0 |
| Multiplicity (# of sites on avg that occur together) | 1.07 |
| Motif File: | file (matrix) reverse opposite |

### Similar de novo motifs found

|  |  |  |  |  |  |  |  |
| --- | --- | --- | --- | --- | --- | --- | --- |
| Rank | Match Score | Redundant Motif | P-value | log P-value | % of Targets | % of Background | Motif file |
| 1 | 0.963 | T C G A A G C T T G A C A G C T T G A C A C T G A G T C A C T G T C G A A C T G T G C A T A C G A G C T | 1e-13 | -30.673353 | 1.00% | 0.40% | motif file (matrix) |
| 2 | 0.980 | C T A G T G C A A C G T T G A C A G C T A G T C A C T G T A G C A C T G C T G A A C T G G T C A | 1e-12 | -29.739452 | 0.52% | 0.13% | motif file (matrix) |
